# Causal association between periodontitis and hypertension: evidence from Mendelian randomization and a randomized controlled trial of non-surgical periodontal therapy

**DOI:** 10.1093/eurheartj/ehz646

**Published:** 2019-09-01

**Authors:** Marta Czesnikiewicz-Guzik, Grzegorz Osmenda, Mateusz Siedlinski, Richard Nosalski, Piotr Pelka, Daniel Nowakowski, Grzegorz Wilk, Tomasz P Mikolajczyk, Agata Schramm-Luc, Aneta Furtak, Pawel Matusik, Joanna Koziol, Miroslaw Drozdz, Eva Munoz-Aguilera, Maciej Tomaszewski, Evangelos Evangelou, Mark Caulfield, Tomasz Grodzicki, Francesco D'Aiuto, Tomasz J Guzik

**Affiliations:** 1 Department of Periodontology and Oral Sciences Research Group, University of Glasgow Dental School, Glasgow, UK; 2 Department of Dental Prophylaxis and Experimental Dentistry, Jagiellonian University Medical College, Krakow, 31-107 Poland; 3 Department of Internal and Agricultural Medicine, Jagiellonian University Medical College, 31-107, Krakow, Poland; 4 Institute of Cardiovascular and Medical Sciences, University of Glasgow, Glasgow, UK; 5 St. Anna’s Hospital, 32-200 Miechow, Poland; 6 Periodontology Unit, UCL Eastman Dental Institute, London, UK; 7 Division of Cardiovascular Sciences, School of Medical Sciences, University of Manchester, Manchester, UK; 8 Department of Epidemiology and Biostatistics, School of Public Health, Imperial College London, UK; 9 William Harvey Research Institute, NIHR Biomedical Research Centre at Barts, Queen Mary University of London, London, UK; 10 Department of Internal Medicine and Gerontology, Jagiellonian University Medical College, 31-107 Krakow, Poland

**Keywords:** Hypertension, Periodontitis, Inflammation, Vascular function, Genetics, Treatment

## Abstract

**Aims:**

Inflammation is an important driver of hypertension. Periodontitis is a chronic inflammatory disease, which could provide a mechanism for pro-hypertensive immune activation, but evidence of a causal relationship in humans is scarce. We aimed to investigate the nature of the association between periodontitis and hypertension.

**Methods and results:**

We performed a two-sample Mendelian randomization analysis in the ∼750 000 UK-Biobank/International Consortium of Blood Pressure-Genome-Wide Association Studies participants using single nucleotide polymorphisms (SNPs) in *SIGLEC5*, *DEFA1A3*, *MTND1P5*, and *LOC107984137* loci GWAS-linked to periodontitis, to ascertain their effect on blood pressure (BP) estimates. This demonstrated a significant relationship between periodontitis-linked SNPs and BP phenotypes. We then performed a randomized intervention trial on the effects of treatment of periodontitis on BP. One hundred and one hypertensive patients with moderate/severe periodontitis were randomized to intensive periodontal treatment (IPT; sub- and supragingival scaling/chlorhexidine; *n* = 50) or control periodontal treatment (CPT; supragingival scaling; *n* = 51) with mean ambulatory 24-h (ABPM) systolic BP (SBP) as primary outcome. Intensive periodontal treatment improved periodontal status at 2 months, compared to CPT. This was accompanied by a substantial reduction in mean SBP in IPT compared to the CPT (mean difference of −11.1 mmHg; 95% CI 6.5–15.8; *P* < 0.001). Systolic BP reduction was correlated to periodontal status improvement. Diastolic BP and endothelial function (flow-mediated dilatation) were also improved by IPT. These cardiovascular changes were accompanied by reductions in circulating IFN-γ and IL-6 as well as activated (CD38+) and immunosenescent (CD57+CD28null) CD8+T cells, previously implicated in hypertension.

**Conclusion:**

A causal relationship between periodontitis and BP was observed providing proof of concept for development of clinical trial in a large cohort of hypertensive patients. ClinicalTrials.gov: NCT02131922.

## Introduction

Hypertension is a common chronic disease impacting 1.4 billion people worldwide, accounts for 10.4 million (9.39–11.5) deaths/year.^1^ Despite the availability of multiple anti-hypertensive agents, 71% of patients fail to achieve blood pressure (BP) below 130/80 mmHg.^2^ Whilst this could be partially explained by poor treatment adherence, persistently elevated BP in these patients, highlights insufficient understanding of the mechanisms of hypertension.

Experimental and observational clinical evidence suggests a prominent role of inflammation in the development of hypertension.^3^ In particular, activation of immune cells has been demonstrated in hypertension.^4–6^ Hypertension is more prevalent in patients with immune-mediated disorders, such as psoriasis, rheumatoid arthritis or systemic lupus erythematosus.^7^ Thus, chronic inflammatory disorders, could provide a substrate for the pro-hypertensive inflammation. 

Periodontitis is one of the commonest inflammatory conditions worldwide, representing the sixth most prevalent condition worldwide^1^^,^^8^ with prevalence of 20–50%.^9^ It is linked to cardiovascular inflammation and endothelial dysfunction.^10^ Therefore, if causally associated, periodontitis could significantly contribute to the global hypertensive burden and interventions targeting oral inflammation would have an important role in the prevention of hypertension and its complications.^8^^,^^11^ Observational evidence suggests that moderate-severe periodontitis is associated with increased odds for hypertension.^12^

Because of this, it is imperative to establish if periodontitis can cause hypertension. Our group has recently shown that immune activation induced by a keystone periodontal pathogen (*Porphyromonas gingivalis*) promotes the development of hypertension in mice.^13^ Small interventional studies concluded that intensive periodontal therapy may lead to BP reduction, although sufficiently powered evidence in well-defined hypertensive cohorts is lacking.^12^

Therefore, we aimed to investigate the nature of the association between periodontitis and hypertension using two experimental approaches: (i) a Mendelian randomization (MR) analysis utilizing genetic, BP data from the UK Biobank, and the International Consortium of Blood Pressure (ICBP)–Genome-Wide Association Studies (GWAS) population and (ii) performing a randomized controlled trial of non-surgical periodontal therapy in hypertensive patients, using 24-h ambulatory BP monitoring as a primary outcome.

## Methods

### Mendelian randomization

Mendelian randomization is one of the methods that can be used to test causal relationship between risk factors and various phenotypes including disease outcomes.^14^ Growing number of single nucleotide polymorphisms (SNPs) that are convincingly associated with certain risk factors (e.g. smoking habits, blood lipid profile, or presence of periodontitis) in GWAS allows researchers to use these SNPs as instrumental variables that approximate lifetime exposure to risk factor and test these SNPs for an association with a phenotype of interest.^14^

A two-sample Mendelian randomization analysis was performed to investigate a causal link between periodontitis and BP. Four SNPs, in *LOC107984137 (rs729876)*, *MTND1P5 (rs16870060)*, *DEFA1A3 (rs2738058)*, and *SIGLEC5 (rs4284742)* loci, previously associated with periodontitis in GWAS,^15^^,^^16^ were used as instrumental variables (IVs) in Mendelian randomization analysis. These SNPs, therefore, were considered as a periodontitis exposure proxy, which were then tested in the context of BP phenotypes. A palindromic rs1537415 SNP in *GLT6D1,* associated with periodontitis in GWAS as well,^17^ was excluded from the analysis due to high allele frequency as well as due to the lack of proxy SNPs in Caucasians. GWAS data on BP, performed by Evangelou *et al*.^18^ from UK-Biobank and ICBP-GWAS (including ∼750 000 participants), were used to extract estimates of association between instrumental variables and systolic BP and diastolic BP (SBP and DBP) and pulse pressure (PP). Additional details are provided in Supplementary material online.

### Randomized clinical trial

We performed a single-centre, parallel-group, randomized study to assess the effect of intensive non-surgical periodontal treatment (IPT; whole mouth subgingival and supragingival scaling of the teeth using also 0.2% chlorhexidine gel) compared with conventional care (supragingival scaling) and a 2-month follow-up. Consecutive patients from general dental practices in Krakow, Poland and from among referrals to the University Dental Clinic in Krakow. Patients who had previously been diagnosed with hypertension (in accordance with ESC/ESH diagnostic criteria^19^ were receiving stable treatment using at least one anti-hypertensive agent, since at least 6 months, and had an office BP of >140/90 mmHg at the time of visit (average of at least three resting measurements)^20^ were enrolled into the study if they also presented with moderate to severe periodontitis (using the Centre for Disease Control–American Association of Periodontology case definitions).^21^^,^^22^ Exclusion criteria included acute and major chronic inflammatory/immune disorders including autoimmune conditions, infections (including tuberculosis, HIV, hepatitis B, and hepatitis C), pulmonary, liver diseases and malignancies (within the last 5 years) as assessed by the examining clinician. Patients who had received treatment with medications known to affect periodontal status were also excluded (phenytoin and cyclosporine). Patients using any form of systemic or local immunosuppression (including steroids) within the previous 6 months were excluded, as were patients with any cause of secondary hypertension.

After identification in general medical practice, all eligible participants have first screened for office BP as well as full dental examination for inclusion into the study. Secondly, the first study visit (baseline) occurred within 3–14 days from the patient identification visit. At baseline, ambulatory 24-h blood pressure monitoring (ABPM), blood samples collection and vascular function assessment were undertaken. ABPM was removed 24-h later in all participants, during the first dental treatment session. All patients who fulfilled criteria and provided informed consent were randomized, after run-in period, 1:1 using a computer-generated table to receive either IPT or CPT. Treatment allocation was concealed in an opaque envelope that was opened on the day of treatment by the treating physician. All other investigators, including cardiovascular physicians, BP nurses, laboratory staff, and staff involved in data collection and analysis remained masked to the treatment allocation.

Intensive periodontal treatment consisted of a single session of whole mouth supragingival and subgingival scaling of the teeth under local anaesthesia with the topical application of 0.2% chlorhexidine gel (PerioKin, UK). CPT consisted of a single session of supragingival scaling of the teeth. All participants received dental hygiene instructions and were then followed up the next day, 7–10 days later, and 2 months after the dental treatment session. Full dental examination and cardiovascular assessments were repeated 2 months after therapy. At the end of the study, patients with CPT received subgingival dental scaling as required. Anti-hypertensive and other medication use by patients was monitored during each visit. Any change would result in termination of patient’s participation in the study.

Average systolic 24-h ambulatory BP at 2 months was the prespecified primary outcome, while vascular function and inflammatory soluble and cellular biomarkers were secondary endpoints. The study was approved by the Jagiellonian University Ethics Committee. All participants provided written informed consent prior to being enrolled into the study. The study was registered with ClinicalTrials.gov: NCT02131922. Additional details are provided in Supplementary material online.

### Office and 24-h ABPM determination

Office BP was measured using Omron M digital BP monitor by a BP clinic nurse. Three measurements after resting in a quiet and temperature-controlled room were performed and average was recorded. ABPM was performed by Department of Internal Medicine ABPM lab using Spacelabs Ultralite 90217 devices in accordance with manufacturer recommendations and in agreement with current ESC/ESH Guidelines.^20^

### Vascular function assessment

Flow-mediated dilatation (FMD) of the brachial artery was used to determine the vascular endothelial function and nitroglycerine-mediated dilatation was used for measuring endothelial-independent vasodilatation. Analysis was performed using Vascular Tools 5 software by two independent vascular technicians masked to the treatment allocation and as previously described.^23^^,^^24^

### Flow cytometric analysis

A panel of circulating blood cell features was performed as previously described.^5^^,^^6^^,^^23^^,^^25^ Additional details are provided in Supplementary material online.

### Plasma cytokine measurements

Blood samples were centrifuged at 400 ×*g* for 10 min. Then, platelet-rich plasma was collected and centrifuged at 1000 ×*g* for 15 min at 4°C. Next, plasma sample without any pelleted cells was collected and stored at −80°C until analysis. Samples were analysed for IFN-γ, IL-1β, IL-6, IL-10, IL-17A, IL-17E, IL-23, IL-33, MIP-3α/CCL20, and TNF-α with Luminex technology using standard kits with magnetic beads (MILLIPLEX MAP Human TH17 Panel—Immunology Multiplex Assay HTH17MAG-14K, Millipore, Merck) and were read on a Luminex 200 machine (Biorad) in accordance with the manufacturer’s instructions.

### Statistical analysis

Analyses were performed with SPSS (ver. 25.0) statistical package unless otherwise stated. MR analysis was performed using MR-PRESSO (Mendelian Randomization Pleiotropy RESidual Sum and Outlier).^26^ Additional causal estimation analyses were performed using inverse-variance weighted, and simple median-based methods using MendelianRandomization package in R (ver. 3.5.1).^27^

Based on previous evidence, the clinical study was powered (80%) with a sample of 50 participants per group to detect a 7 mmHg difference in BP between study groups and a 13 mmHg standard deviation of the change in SBP.^28–31^ All the analyses were performed based on the intention-to-treat principle, per-protocol analyses for the outcomes reported are included. Means and 95% confidence intervals (95% CIs) of continuous variables are presented according to treatment groups. Categorical variables count and percentages are presented according to the treatment group and values were tested using the χ^2^ test. In the discovery analyses, cytokines and cell types were compared using the paired *t*-test for each of the treatment groups separately. Between group differences and differences from baseline to 2-month of follow-up were tested with the use of repeated-measures analysis of variance (ANOVA) with interaction term between group and time defined by two visits. We used one-sided tests for analysis of cytokines and cell types based on hypothesis from previous studies.^28^ Correlation analyses between the BP outcomes and changes in dental parameters were performed using Spearman Rank tests. Mediation analysis was performed using mediation package in R^32^^,^^33^ and tested average causal mediation effect of treatment group on change in SBP that is due to the change in periodontal pocket depth (PPD). *P*-values <0.05 were considered significant.

## Results

### Mendelian randomization links hypertension to periodontitis

Mendelian randomization utilizes genetic variants that serve as proxy measures for modifiable risk factors to allow estimation of the causal influence of the modifiable risk factor in question. We, therefore, used four SNPs, in *LOC107984137*, *MTND1P5*, *DEFA1A3*, and *SIGLEC5* loci, previously associated with periodontitis in GWAS, as instruments for making causal inferences about the role of periodontitis in human BP regulation. All studied SNPs showed concordant effect direction i.e. the same allele was associated with both increased risk for periodontitis and increased level of BP index (Supplementary material online, *Table S1* and *Figure S1*). MR-PRESSO analysis did not identify significant horizontal pleiotropic variants and demonstrated a significant effect of periodontitis on all three BP indexes. Median-based and inverse-variance weighted approaches detected nominally significant causal association between periodontitis-linked SNPs and SBP or PP (*Table 1*).

**Table 1:** Results of Mendelian randomization analysis in the combined UK Biobank and International Consortium of Blood Pressure–Genome-Wide Association Studies populations

Phenotype	MR-PRESSO			Median			IVW (inverse-variance weighted)		
	Causal estimate	SD	*P*	Causal estimate	SE	*P*	Causal estimate	SE	*P*
Systolic blood pressure (SBP)	0.177	0.014	0.001	0.177	0.084	0.036	0.177	0.072	0.013
Diastolic blood pressure (DBP)	0.069	0.012	0.011	0.076	0.048	0.114	0.069	0.041	0.093
Pulse pressure (PP)	0.110	0.015	0.005	0.115	0.057	0.044	0.110	0.049	0.023

### Clinical study

**Figure 1:**
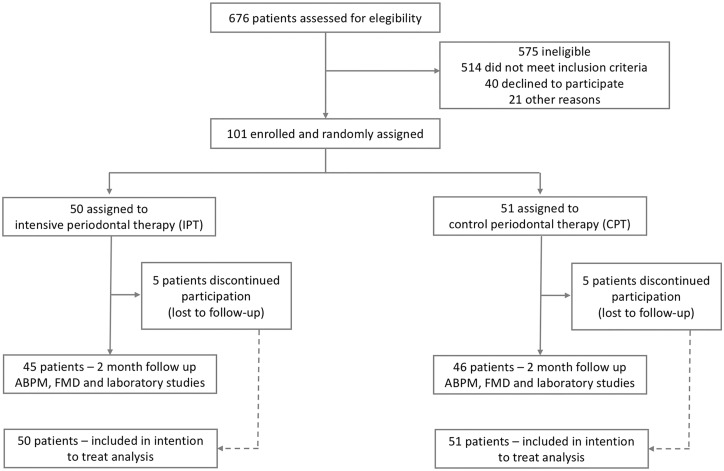
A flowchart of clinical study profile.

About 101 consecutive patients were randomized to IPT or CPT control group between 10 December 2009 and 5 January 2015. Whilst office BP of all patients exceeded 140/90 mmHg in all participants, the whole group active period ABPM was 137 ± 15/86 ± 10 mmHg. Both study groups did not significantly differ in any of the medical or dental characteristics at baseline (*Table 2*). Fifty patients were assigned to IPT, while 51 were assigned to control treatment (*Figure 1*). Notably, in both groups, five patients did not reach the 2-month time point and were lost to follow-up (*Figure 1*). No serious adverse events were reported.

**Table 2:** Baseline characteristics of the study population

	Control periodontal treatment (CPT; *n* = 51)	Intensive periodontal treatment (IPT; *n* = 50)
Age (years)	56 (54–58)	53 (50–56)
Sex, *n* (%)		
Male	31 (60)	26 (52)
Female	20 (40)	24 (48)
BMI	29 (28.1–30.7)	28 (26.8–29.2)
Smoking, *n* (%)		
Never	26 (51)	26 (51)
Current	15 (29)	17 (34)
Past	10 (20)	7 (14)
Diabetes mellitus, *n* (%)		
Type 1	0	0
Type 2/glucose intolerance	6 (11)	4 (8)
Office SBP (mmHg)	154 (150.6–158.9)	155 (152.4–158.5)
Office DBP (mmHg)	89 (85–92)	92 (89–95)
Ambulatory 24 h BP		
Average SBP (mmHg)	132 (127–136)	135 (130–138)
Average DBP (mmHg)	80 (77–82)	84 (80–86)
Heart rate (b.p.m.)	72 (69–74)	72 (69–75)
Activity SBP (mmHg)	136 (131–140)	138 (135–142)
Activity DBP (mmHg)	83 (80–86)	87 (85–90)
Night SBP (mmHg)	119 (114–124)	121 (117–126)
Night DBP (mmHg)	70 (67–73)	71 (68–74)
Total cholesterol (mmol/L)	5.3 (5.0–5.6)	5.8 (5.5–6.1)
TG (mmol/L)	1.8 (1.5–2.2)	1.7 (1.5–2.0)
LDL cholesterol (mmol/L)	3.2 (2.9–3.4)	3.6 (3.4–3.9)
HDL cholesterol (mmol/L)	1.3 (1.2–1.4)	1.4 (1.3–1.5)
Creatinine (µmol/L)	92 (89.1–96.7)	88 (84.2–91.5)
Fasting Glucose (mmol/L)	5.7 (5.5–6.1)	5.4 (5.2–5.7)
White blood cells (1000/mm^3^)^a^	6.7 (6.1–7.3)	6.1 (5.7–6.5)
Mean periodontal probing depth (mm)	2.9 (2.8–3.1)	2.9 (2.8–3.1)
Mean CAL (mm)	3.7 (3.4–4.0)	3.8 (3.5–4.1)
Mean CIPTN	3.2 (3.1–3.4)	3.1 (2.9–3.3)
Number of teeth	21 (18.7–22)	22 (19.7–23.1)
Number of anti-hypertensives	2.2 (1.8–2.5)	2.1 (1.8–2.4)
Beta-blocker, *n* (%)	22 (43)	20 (40)
Calcium channel blocker, *n* (%)	19 (37)	16 (32)
ACR-I/ARB, *n* (%)	38 (75)	37 (74)
Alpha-blocker, *n* (%)	4 (8)	4 (8)
Diuretic, *n* (%)	29 (56)	26 (52)
HMG-CoA inhibitors, *n* (%)	16 (31)	13 (26)

a Data are averages with 95% CI or patient numbers with percentages in brackets. All differences in characteristics between groups were non-significant.

ACE, angiotensin-converting enzyme; ARB, angiotensin receptor blockers; BMI, body mass index; CAL, clinical attachment loss; CIPTN, community periodontal index of treatment needs; HMG-CoA, β-hydroxy β-methylglutaryl-coenzyme A; TG, triglycerides.

### Effects of periodontal treatment on blood pressure

**Figure 2:**
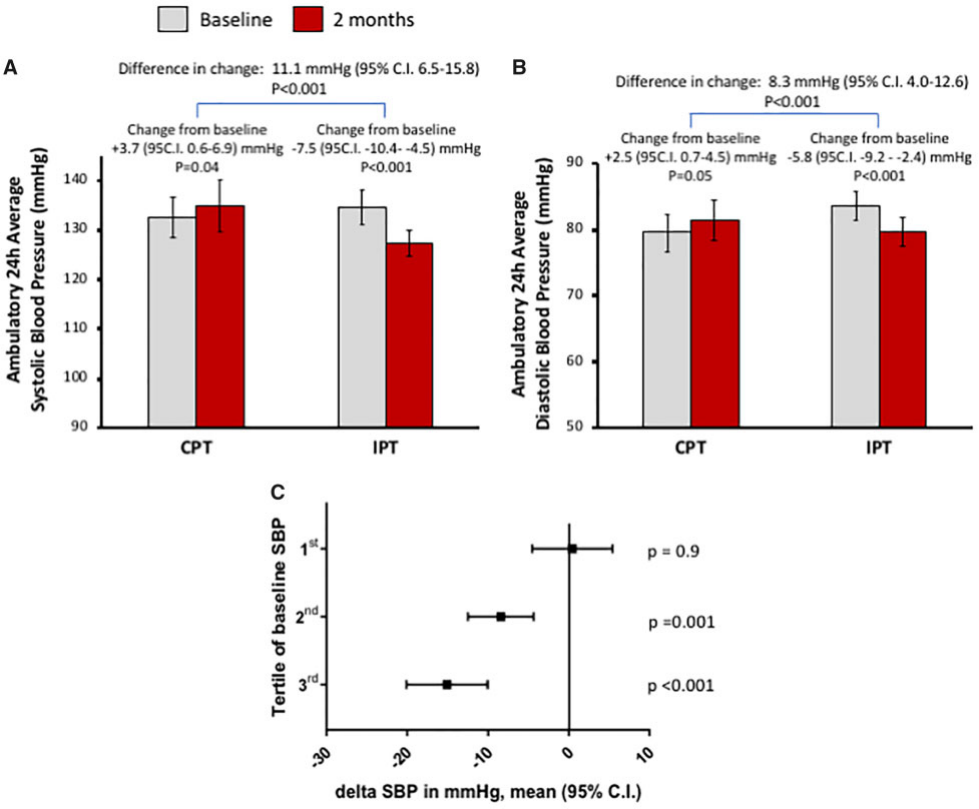
Effects of conventional periodontal treatment and intensive periodontal treatment on blood pressure. Changes of 24-h average systolic (*A*) and diastolic (*B*) blood pressure between baseline and 2 months following control periodontal treatment or intensive periodontal treatment are reported as mean ± 95% confidence interval. Subsequently, difference in change was calculated between randomization groups. (*C*) Relationship between baseline systolic blood pressure and the effect of intensive periodontal treatment on blood pressure reduction. Patients were divided in tertiles of baseline ambulatory 24-h blood pressure monitoring measured systolic blood pressure and change of systolic blood pressure between baseline and follow-up (delta systolic blood pressure) was analysed and presented as mean with 95% confidence interval. Ambulatory 24-h blood pressure monitoring Tertile 1: <130 mmHg; Tertile 2: 131–138 mmHg; and Tertile 3 >138 mmHg.

Intensive periodontal treatment caused a significant reduction of average 24-h SBP (*Figure 2A*) as well as DBP (*Figure 2B*) by 7.5 and 5.8 mmHg, respectively. Interestingly, in the control treatment group a borderline significant increases of BP values were observed, leading to the difference in change of SBP of 11.1 mmHg (95% CI 6.5–15.8) and DBP of 8.3 mmHg (95% CI 3.98–12.6) between CPT and IPT groups (*Figure 2A* and *B*). These changes in BP were not accompanied by any change in average daily heart rate (72 ± 10 vs. 71.6 ± 9 b.p.m.; *P* = 0.3). We also analysed anti-hypertensive effect of IPT in relation to baseline BP showing, as expected, that the degree of SBP reduction was dependent on baseline SBP value (*Figure 2C*). Significant systolic, diastolic, and PP reductions were observed both in daytime (activity) and during the nighttime (Supplementary material online, *Table S2*). We have also performed a subgroup analysis to determine if any of patient subgroups benefited the most. While no difference was observed depending on sex, initial periodontal status, or number of anti-hypertensive medications, a significant effect (*P*-value for interaction < 0.05) was evident for age and borderline effect for body mass index, indicating that periodontitis might have stronger link to hypertension in younger individuals. (Supplementary material online, *Figure S3*).

### Effects of periodontal treatment on vascular function

Intensive periodontal treatment participants presented with increased FMD at 2 months after therapy compared to CPT [difference of −1.72% (95% CI −3.1 to −0.4); *P* < 0.01; Supplementary material online, *Figure S2A*]. Non-endothelium dependent, nitroglycerine-induced vasodilatation did not change between study groups (Supplementary material online, *Figure S2B*).

### Dental status changes

**Figure 3:**
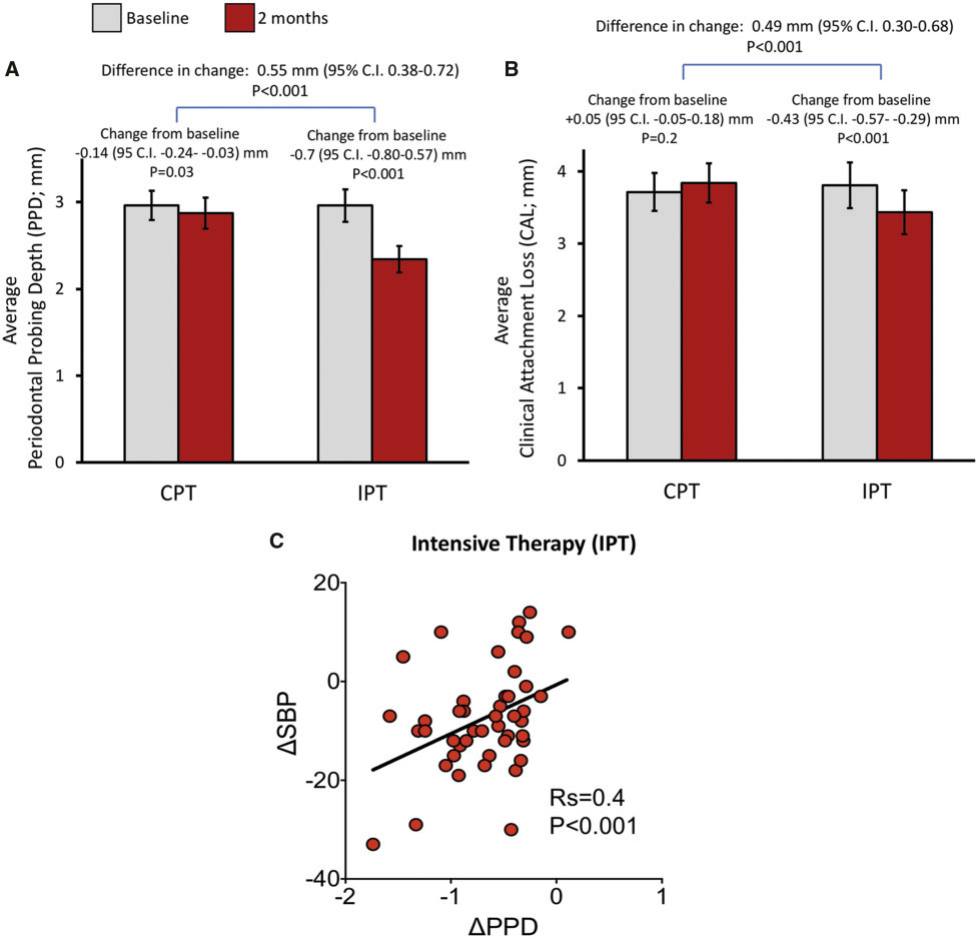
Effects of control periodontal treatment and intensive periodontal treatment on periodontal status and its relationship to blood pressure improvement. Changes of periodontal pocket depth (*A)* and clinical attachment loss (*B*) were monitored between baseline and 2 months following control periodontal treatment or intensive periodontal treatment and are reported as mean ± 95% confidence interval along with difference in change calculated between randomization groups (with 95% confidence interval). (*C*) Spearman correlation between improvement of systolic blood pressure and change of periodontal pocket depth in individual patients.

Intensive periodontal treatment produced a substantial improvement in periodontal health of all participants when compared to CPT patients [periodontal probing pocket depth difference of 0.55 mm, 95% CI 0.38–0.72, *P* < 0.001; *Figure 3A*]. Similar differences were observed in average periodontal clinical attachment level (*Figure 3B*). Notably, the degree of individual BP change at 2 months was positively correlated with the change of periodontal probing depth (*Figure 3C*) in the IPT group. This correlation remained statistically significant after correction for the baseline SBP value (*R*s = 0.34; *P* < 0.05) or when all studied participants were taken into account (*R*s = 0.46; *P* < 0.001). Moreover, to test whether changes in periodontal status mediate the benefits of IPT on BP we performed mediation analysis. This demonstrated a significant average direct effect of treatment group on change in SBP [estimate = 8.92 (95% CI 3.68–14.29), *P* = 0.001] and a significant average causal mediation effect of treatment group on change in SBP that is due to change in PPD [estimate = 3.59 (95% CI 0.52–7.10), *P* = 0.021; proportion of total effect due to mediation = 0.29 (95% CI 0.04–0.61), *P* = 0.021].

### Cell and circulating inflammatory mediators

Luminex analyses confirmed that IPT produced modest but statistically significant reductions in plasma levels of IL-6, IFN-γ, IL-17A, and TNF-α (*Table 3*). The differences remained significant in ANOVA only in relation to IFN-γ (CPT change: −2.2 ± 4.7, IPT change: −12.6 ± 2.8); difference in change: 10.4 pg/mL and IL-6 (CPT change: 0.3 ± 2.4, IPT change: −5.7 ± 2.2); difference in changes: 6.0 pg/mL. No differences were noted in the other circulating biomarkers.

**Table 3:** Changes of systemic plasma cytokine levels in subjects following control periodontal treatment and intensive periodontal treatment

Cytokine	CPT					IPT				
	Baseline	2 months	2 months—baseline	*P*-value	Adjusted *P*-value (FDR)	Baseline	2 months	2 months—baseline	*P*-value	Adjusted *P*-value (FDR)
	Mean (95% CI)	Mean (95% CI)	Delta (95% CI)			Mean (95% CI)	Mean 95% CI)	Delta (95% CI)		
IFNγ (pg/mL)	62.74 (50.0–75.5)	60.54 (48.2–72.9)	−2.20 (−11.9 to 7.6)	0.322	0.402	60.73 (49.9–71.6)	48.17 (38.7–57.5)	−12.56 (−18.3 to 6.8)	0.0001	**0.001**
IL17A (pg/mL)	35.87 (28.8–42.9)	33.38 (26.2–40.5)	−2.47 (−7.4 to 2.4)	0.152	0.253	35.68 (27.9–43.5)	28.72 ± 3.3 (21.9–35.5)	−6.96 (−11.6 to −2.4	0.002	**0.012**
TNF-α (pg/mL)	29.8 (25.5–34.1)	27.9 (23.4–32.4)	−1.89 (−5.2 to 1.4)	0.121	0.243	27.9 (23.6–32.2)	23.77 (20.3–27.3)	−4.13 (−7.3 to −1.0)	0.006	**0.020**
IL6 (pg/mL)	26.51 (19.8–33.2)	26.78 (19.4–34.2)	0.28 (−4.8 to 5.4)	0.455	0.487	25.47 (16.0–34.9)	19.78 (13.0–16.6)	−5.69 (−10.3 to −1.1)	0.009	**0.023**
IL23 (ng/mL)	4.64 (3.2–6.0)	3.9 (2.7–5.1)	−0.736 (−1.4 to −0.5)	0.019	0.094	3.93 (3.0–4.9)	3.46 (2.6–4.4)	−0.46 (−1.1 to 0.1)	0.058	0.117
CCL20 (pg/mL)	47.73 (40.9–54.5)	55.54 (45.7–65.4)	7.81 (−2.4 to 18.0)	0.064	0.160	59.14 (37.8–80.5)	45.81 (38.8–52.8)	−13.33 (−32.2 to 5.6)	0.079	0.122
IL1β (pg/mL)	25.2 (20.9–29.5)	21.42 (16.4–26.5)	−3.78 (−7.0 to −0.5)	0.013	0.094	23.64 (18.3–28.9)	21.61 (16.6–26.6)	−2.03 (−5.0 to 0.9)	0.086	0.122
IL10 (pg/mL)	6.78 (5.1–8.5)	6.8 (4.9–8.7)	0.02 (−1.3 to 1.4)	0.487	0.487	6.30 (4.3–8.3)	7.02 (5.2–8.9)	0.72 (−0.4 to 1.9)	0.105	0.131
IL33 (pg/mL)	101.3 (82.1–120.5)	89.09 (66.9–109.3)	−12.23 (−25.5 to 1.0)	0.034	0.115	86.62 (71.0–102.2)	81.01 (64.9–97.2)	−5.61 (−17.8 to 6.6)	0.175	0.195
IL17E (pg/mL)	2.43 (1.5–3.4)	2.28 (1.4–3.2)	−0.15 (−0.6 to 0.3)	0.235	0.335	2.02 (1.2–2.8)	1.94 (1.2–2.7)	−0.072 (−0.6 to 0.5)	0.399	0.399

Significant values are underlined and FDR - values are given in bold.

**Figure 4:**
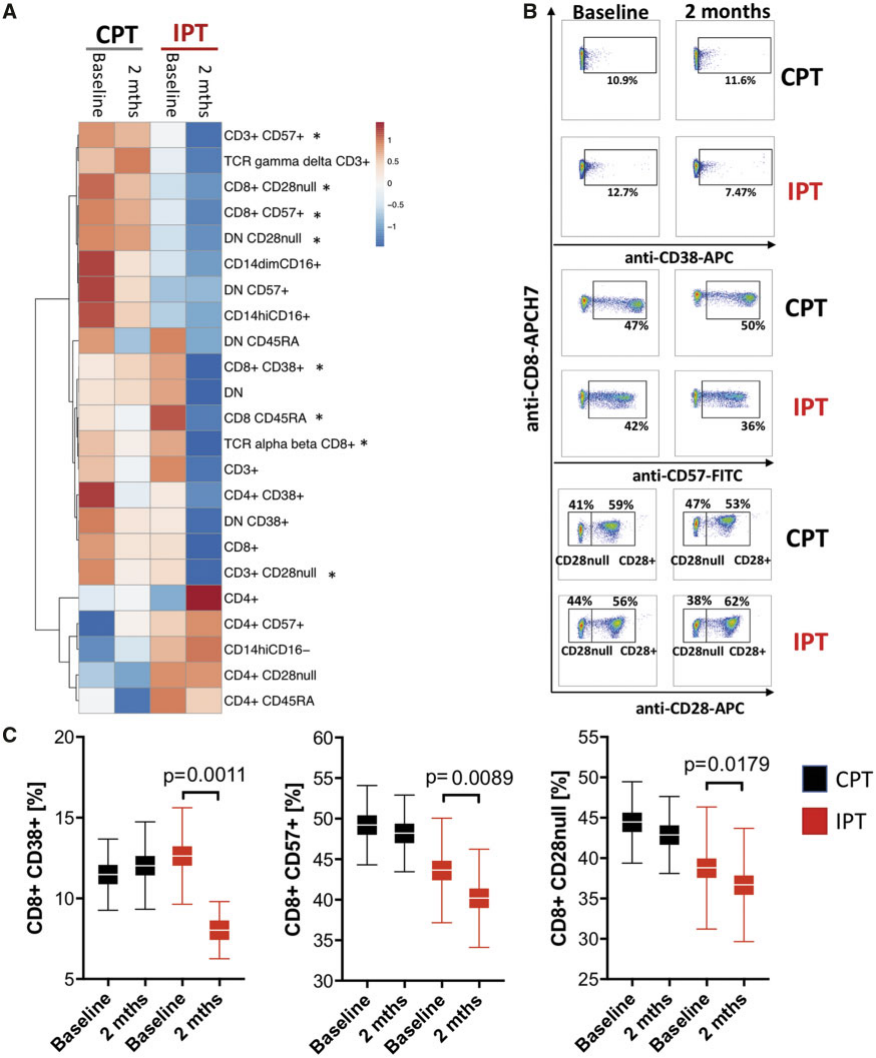
Effects of control periodontal treatment and intensive periodontal treatment on selected T lymphocyte and monocyte populations. (*A*) Heatmap presenting 23 cell subpopulations with ratio of baseline- and post-treatment levels between control periodontal treatment and intensive periodontal treatment patients. Cell types were selected on the basis of previous reports of significance for hypertension and vascular regulation. Colour of each square corresponds to a baseline and follow-up (2 months) means for intensive periodontal treatment and control periodontal treatment groups of each analysed cell type. Differences in absolute values that were significant (*P* < 0.05) after FDR correction in intensive periodontal treatment but not control periodontal treatment in our discovery analysis are marked with asterisk. Heatmap was generated using ClustVis software. Examples of flow cytometry (*B*) and average values (*C*) detecting cell types of activated (CD38) and immunosenescent (CD28null, CD57+) CD8+T cells between baseline and 2 months post-treatment in control periodontal treatment and intensive periodontal treatment groups. *P < 0.05 vs. baseline; two-way analysis of variance.

Whilst no changes in pro-inflammatory monocytes or CD4+ T cells were observed in IPT, we observed a substantial reduction of activated CD8 cells characterized by expression of CD38+ cells in the IPT but not CPT group (*Figure 4*). This reduction remained statistically significant after correction for multiple comparisons (FDR) and in two-way ANOVA analyses [CPT change: 0.56 ± 1.27, IPT change: −4.60 ± 1.39; difference in changes: 5.16 (95% CI 1.39–8.93), *Figure 4*]. Moreover, a reduction in circulating immunosenescent CD8+ CD28nullCD57+ cells between study groups was observed in IPT but not CPT group (*Figure 4A–C*).

## Discussion

In the present study, we report evidence of a causal relationship between periodontitis and hypertension using two complementary and independent research methods. Firstly a two-sample MR approach using a large sample population (∼750 000 participants) and non-palindromic SNPs analysis linking loci that were previously GWAS-associated to periodontitis with hypertension. The association between periodontitis and hypertension was also supported by a randomized controlled trial of non-surgical periodontal therapy. Improvement of periodontal status, following IPT, was directly related to the 24-h BP profile improvement. These effects on BP were accompanied by reduction of systemic pro-hypertensive cytokine levels ([IFN-γ, IL-6, IL-17A, and TNF-α), as well as activated (CD38+) and immunosenescent dysregulated CD57+/CD28null CD8+ T cells, that were previously linked to hypertension.^34^

This is the first study using a two-sample MR to gain inferences about a causal relationship between periodontitis and BP indexes. While recent publications of GWAS studies have helped in identifying possible genetic tools,^15^^,^^16^ a low number of available instrumental variables remains a limitation of this approach. Depending on the calculation method, heritability estimates of periodontitis are highly variable and included instrumental variables likely explain small proportion of genetic background of periodontitis.^35^ Nevertheless, it did allow for successful identification of the links between periodontitis-linked SNPs and systolic, diastolic, and PP using a number of independent Mendelian randomization approaches. This indicates that in the future, this genetic instrument can be very valuable to address the link between periodontitis and hard cardiovascular endpoints.

In the studies used to generate the Mendelian randomization results, information on hard cardiovascular outcomes are available. This is important as the link between periodontitis and risk cardiovascular disease remains a topic of extreme interest. Genetic tool used in this study, can be useful in future investigations of hard cardiovascular endpoints.

To the best of our knowledge, our work is the first attempt to address the question of whether non-surgical periodontal treatment can reduce BP in hypertensive patients with ambulatory 24-h BP monitoring as the primary outcome. The use of 24-h ABPM as a primary endpoint in our study is of particular importance for consistency and validity of results in modestly sized trials.^36^ Out of a total of 12 interventional studies performed to date in patients with periodontitis that have reported BP values, only one addressed the question of BP reduction as a primary outcome,^12^ however, this involved patients with high normal BP rather than those with hypertension. Zhou *et al.*^37^ reported an absolute difference of office SBP of 12.57 mmHg, 95% CI 10.45–14.69, *P* < 0.05 between treatment arms. In majority of other interventional studies of sufficient size, including our recent clinical trials, where BP was reported, uncontrolled hypertension was an exclusion criterion.^10^^,^^28^

In our study, IPT was associated with a 7.5 ± 10 mmHg reduction of BP. This could represent an important health change linked to prevention of hypertension complications. A recent meta-analysis of hypertensive randomized clinical trials reported that a 10-mmHg reduction in systolic and/or 5-mmHg reduction in DBP resulted in a 25–30% reduction of cardiovascular events including stroke and heart failure.^38^ Our results are consistent with recent large cross-sectional NHANES survey of 11 753 participants that showed that good periodontal health is associated with better SBP profile during anti-hypertensive therapy by about 2.3–3 mmHg and with lower odds of anti-hypertensive treatment failure.^39^

In this study, we observed improvements in endothelial function after periodontal therapy. As endothelial function is one of the key mechanisms for vascular risk in hypertension, our observations linking it to periodontal inflammation is of primary importance. This is consistent with the previous observations that periodontitis is associated with endothelial dysfunction and that the latter is improved by IPT.^10^ More importantly, such improvement, in hypertensive patients was already observed 2 months after IPT, earlier than in other cardiovascular risk populations.^10^ We have not measured pulse wave velocity or vascular stiffness, as our follow-up period was likely too short to observe significant change. In future, longer, studies it may represent a valuable outcome providing additional insights into vascular biology of periodontitis.

Periodontitis is associated with systemic inflammation evidenced by high C-reactive protein/high asymmetric dimethylarginine levels,^40^ as well as circulating cytokines and adipokines.^28^ In addition, as white blood cell counts have been linked to periodontitis,^41^ we also investigated cellular signature of inflammation. Reductions of circulating activated and dysregulated CD8 cells of immunosenescent phenotype observed in this study are of interest,^42^ as patients with hypertension show an increased presence of immunosenescent, pro-inflammatory, cytotoxic CD8+ T cells characterized by presence of CD57+ and lack of CD28 (CD28null).^34^ Interestingly these cells are an important source of TNF-α and IFN-γ and they have been shown to infiltrate kidneys and vessels in hypertensive patients, through which they can contribute to development of the disease. Moreover, we have also recently demonstrated that these cells (CD57_CD28null) are first to be recruited to sites of vascular injury in humans which makes them particularly important from the perspective of vascular function regulation.^25^ However number of conditions can affect development of these immunosenescent cells^34^ and, while interesting, extent of changes observed here was modest, and does not prove their causal role linking periodontitis to hypertension.

While we have measured a number of pro-and anti-inflammatory cytokines, particularly notable reductions were observed in pro-inflammatory cytokines which have been shown to contribute to hypertension by previous studies. This includes TNF-α,^43^ IFN-γ,^44^ IL-17A,^45^ and IL-6.^46^ In particular, IFN-γ has been associated with vascular and BP phenotypes.^34^^,^^44^^,^^47^^,^^48^

Interestingly, these systemic changes were observed in our patients who were characterized by moderate to severe level of periodontitis. Notably, average whole mouth periodontal probing depth was lower than in some of the previous trials which recruited patients with more severe forms of periodontitis. This was likely linked to our model of patient ascertainment from general medical practices rather than solely periodontal clinics. We have clearly observed that in such patients, even after a single treatment visit periodontal status remains improved after 2 months with reduction of systemic inflammatory markers. In longer-term observations, the effects of initial therapy are often obscured by poor adherence of patients regarding domiciliary dental hygiene routines. Our study represents a unique proof-of-concept which could well serve for the design of a larger, multi-centre and longer follow-up trial.

One of the surprising observations of the current study was an increase of ABPM BP in control treatment patients during the follow-up. This is consistent with recent observation in a high normal BP group of patients.^37^ At 2 months following conventional treatment, we did not observe significant changes of inflammatory cytokines. Other factors linked to continued periodontitis could also contribute to worsening of BP control in this group of patients. Such change could be related to worse treatment adherence in this group of patients, although it is important to note that we studied patients with long-term chronic hypertension who have already used stable treatment before inclusion into the study.

### Limitations

Our clinical study should be considered as proof of concept and needs to be confirmed in a large cohort of hypertensive patients. Firstly, the population included in the trial cannot be considered as having resistant hypertension, but simply as an insufficiently controlled BP. In order to ensure that changes of treatment will not be a major cause for BP alterations, patients who required a change of anti-hypertensive therapy during the trial were excluded. While in a 2-month time of follow-up this was a rare event, it might be an important consideration for future long-term studies, as may either preferentially exclude or include patients particularly prone to inflammatory mechanism. In line with this, effects of periodontal therapy on medication adherence need to be carefully monitored as it is highly important for addressing the effect of diverse therapies on BP outcomes. Similarly, while number of patients studied was sufficient to show effects on primary outcome, inclusion of more participants would have strengthened the study results given the variability of the response to therapy. While our study provides valuable proof of concept, a longer follow-up of 6 or 12 months would be needed to allow for therapeutic recommendations and conclusions. Finally, while not significant, the intensive treatment group compared to standard treatment was characterized by numerically higher 24-h blood pressure. While this difference is not significant, we have to remain cautious that it might have affected BP outcome.

In conclusion, the present study supports a causal relationship between periodontitis and hypertension. While the mechanisms of this relationship warrant further investigation, we have provided genetic and experimental evidence that periodontitis is linked to hypertension. These preliminary results need to be confirmed in a large cohort of hypertensive patients as they may represent a novel non-pharmacological intervention for the management of hypertension.

## Supplementary Material

ehz646_Supplementary_Materials

## Notes

**See page 3471 for the editorial comment on this article (doi: 10.1093/eurheartj/ehz729)**
