# Supplementary material for: Causal association between periodontitis and hypertension: evidence from Mendelian randomization and a randomized controlled trial of non-surgical periodontal therapy
Source: Eur Heart J. 2019 Sep 1;40(42):3459–70. doi: 10.1093/eurheartj/ehz646 (PMC6837161; doi:10.1093/eurheartj/ehz646)
Supplement: ehz646_Supplementary_Materials [file ehz646_supplementary_materials.pdf]

Causal association between periodontitis and hypertension: Evidence from Mendelian Randomization and a Randomized Controlled Trial of non-surgical periodontal therapy.

Supplementary data

M. Czesnikiewicz-Guzik[1,2], G. Osmenda[3], M. Siedlinski[3,4], R. Nosalski[3,4], J. Pelka[2], D. Nowakowski[2], G. Wilk[3], T. P. Mikolajczyk[3], A. Schramm-Luc[3], A. Furtak[2], P. Matusik[3], Koziol J. [3], M. Drozdz[5] E. Munoz Aguilera[6], M. Tomaszewski[7], E. Evangelou[8], M. Caulfield[9], T. Grodzicki[10], F. D'Aiuto [6], TJ Guzik [3,4]

Extended Methods:

Mendelian Randomization

Mendelian randomization is one of the methods that can be used to test causal relationship between risk factors and various phenotypes including disease outcomes using genetic variation as a natural experiment. Growing number of SNPs that are convincingly associated with certain risk factors (e.g. smoking habits, blood lipid profile or presence of periodontitis) in GWAS allows researchers to use these SNPs as instrumental variables that approximate lifetime exposure to risk factor and test these SNPs for an association with a phenotype of interest. There are three key assumptions that MR analysis must meet to be valid:1. Relevance – selected SNPs associate with the risk factor, 2-Independence – lack of unmeasured confounders of the association between SNP and the outcome 3. Exclusion restriction-SNPs affect the outcome exclusively via their effect on selected risk factor [1]. Several statistical tests were developed in order to provide valid estimates of MR analysis. Inverse variance weighted method provides unbiased results when the 3rd assumption is met i.e. no horizontal pleiotropic variants are included in the analysis. Horizontal pleiotropy occurs when a genetic variant affects hypertension both through periodontitis and mechanisms other than periodontitis. Since it is often difficult to ensure lack of horizontal pleiotropic variants in the study, other methods are often used. For example, median-based methods proved valid estimates when at least half of the tested variants have no pleiotropic effects [1]. A recently developed Mendelian randomization pleiotropy residual sum and outlier (MR-PRESSO) test aims to identify pleiotropic variants in order to correct results of MR analysis accordingly [2].

A two-sample mendelian randomization (MR) analysis was performed to investigate a causal link between periodontitis and blood pressure. Four SNPs, in *LOC107984137 (rs729876)*, *MTND1P5 (rs16870060)*, *DEFA1A3 (rs2738058)* and *SIGLEC5 (rs4284742) loci*, previously associated (with p value $<5x10^{-8}$) with periodontitis in GWAS [3, 4], were used as instrumental variables (IVs) in Mendelian Randomization analysis. These SNPs, were then used as a periodontitis' exposure proxy, which were then tested in the context of blood pressure phenotypes. A palindromic rs1537415 SNP in *GLT6D1,* associated with periodontitis in GWAS as well [5], was excluded from the analysis due to high allele frequency as well as due to the

lack of proxy SNPs in Caucasians. For rs1537415 strand and allele alignment were deemed unreliable between different studies (i.e. original GWAS for periodontitis and ICBP+UK Biobank GWAS for blood pressure traits).

GWAS data on blood pressure, performed by Evangelou et al. [6] from UK-Biobank and ICBP-GWAS (including ~750,000 participants), were used to extract estimates of association between IVs and systolic, diastolic blood pressure (SBP, DBP) and pulse pressure (PP). As indicated in the original studies, off-treatment blood pressure values for treated individuals were imputed by adding 15mmHg to measured SBP and 10mmHg to measured DBP for all treated individuals. [6, 7]

Randomized Clinical Trial

We performed a single-center, parallel-group, randomized study to assess the effect of intensive non-surgical periodontal therapy (IPT; whole mouth subgingival and supragingival scaling of the teeth using also 0.2% chlorhexidine gel) compared with conventional care (supragingival scaling) and a 2 months follow-up. Consecutive patients from general dental practices in Krakow, Poland and from among referrals to the University Dental Clinic in Krakow. Patients who had previously been diagnosed with hypertension (in accordance with ESC/ESH diagnostic criteria [8], were receiving stable treatment using at least 1 anti-hypertensive agent, since at least 6 months, and had an office blood pressure of >140/90 mmHg at the time of visit (average of at least 3 resting measurements) [9] were enrolled into the study if they also presented with moderate to severe periodontitis (using the CDC–AAP case definitions) [10, 11]. In brief, moderate periodontitis was defined as ≥2 interproximal sites with AL ≥4 mm (not on same tooth), or ≥2 interproximal sites with PD ≥5 mm (not on same tooth)and severe periodontitis as≥2 interproximal sites with AL ≥6 mm (not on same tooth) and ≥1 interproximal site with PD ≥5 mm Exclusion criteria included acute and major chronic inflammatory/immune disorders including autoimmune conditions, infections (including tuberculosis, HIV, hepatitis B and C), pulmonary, liver diseases and malignancies (within the last 5 years) and assessed by the examining clinician. Patients who had received treatment with medications known to affect periodontal status were also excluded (phenytoin and ciclosporin). Patient with furcation involvement were also excluded. Patients using any form

of systemic or local immunosuppression (including steroids) within the previous 6 months were excluded, as were patients with any cause of secondary hypertension.

After identification in general medical practice all eligible participants were first screened for office blood pressure as well as full dental examination for inclusion into the study. Secondly, the 1st study visit (baseline) occurred within 3-14 days from the patient identification visit. At baseline, ambulatory 24h blood pressure monitoring (ABPM), blood samples collection and vascular function assessment were undertaken. ABPM was removed 24 hours later in all participants, during the first dental treatment session. All patients who fulfilled criteria and provided informed consent were randomized, after run-in period, 1:1 using a computer-generated table to receive either IPT or CPT. Treatment allocation was concealed in an opaque envelope that was opened on the day of treatment by the treating physician. All other investigators, including cardiovascular physicians, blood pressure nurses, laboratory staff as well as staff involved in data collection and analysis remained masked to the treatment allocation.

IPT consisted of a single session of whole mouth supragingival and subgingival scaling of the teeth under local anesthesia with the topical application of 0.2% chlorhexidine gel (PerioKin; UK). CPT consisted of a single session of supragingival scaling of the teeth. All participants received dental hygiene instructions and were then followed up the next day, 7-10 days later and 2 months after the dental treatment session. Full dental examination and cardiovascular assessments were repeated 2 months after therapy. At the end of the study patients with CPT received subgingival dental scaling as required. None of the patients in the study required additional session of dental treatment during the study. Patient’s report of their drug adherence was recorded during each visit. Patients were asked to bring their medications to each visit and were carefully interviewed regarding adherence. Patient anti-hypertensive medications were provided as standard of care. Any change of medication/dosage resulted in exclusion from the study and this happened in 2 patients (4%) in intensive group and 3 patients (5.8%) in conventional therapy group in patients who were subsequently lost to follow-up. In summary, no difference in adherence was observed using this approach.

Average 24h ambulatory blood pressure at 2 months was the prespecified primary outcome, while vascular function and inflammatory soluble and cellular biomarkers were secondary endpoints. The study was approved by the Jagiellonian University Ethics Committee. All

participants provided written informed consent prior to being enrolled into the study The study was registered with ClinicalTrials.gov - NCT02131922

Office and 24h ABPM determination

Office blood pressure was measured using Omron M digital blood pressure monitor by a blood pressure clinic nurse. Three measurements after resting in a quiet and temperature-controlled room were performed and average was recorded. ABPM was performed using Spacelabs Ultralite 90217 devices in accordance with manufacturer recommendations and in agreement with current ESC/ESH Guidelines.[9] ABPM was analyzed by a central ABPM lab of the Department of Internal Medicine, J Dietl Hospital in Krakow, Poland. Technicians as well as investigators analyzing the results were blinded to study group allocation and had no contact with patients.

Vascular Function Assessment

Flow-mediated dilatation (FMD) of the brachial artery was used to determine the vascular endothelial function and NMD (nitroglycerine-mediated dilatation) was used for measuring endothelial-independent vasodilatation. Analysis was performed analyzed using Vascular Tools 5 software by 2 independent vascular technicians masked to the treatment allocation and as previously described.[12, 13]

Flow cytometric analysis

Blood samples were collected from patients into ethylenediaminetetraacetic acid (EDTA) tubes (BD Vacutainer). Whole blood was centrifuged to separate plasma, then Peripheral blood mononuclear cells (PBMC) were isolated by standard gradient centrifugation using Lymphocyte Separation Medium (LSM) 1077 (PAA Laboratories GmbH, Austria). PBMCs were further suspended in phosphate-buffered saline (PBS) containing 1% heat-inactivated fetal bovine serum (FBS) (Gibco, Life Technologies, USA) and were used immediately after isolation. A total of 0.5x106 PBMCs were stained for 20 minutes with fluorochrome-conjugated monoclonal antibodies. The following monoclonal antibodies were used: anti-CD3-PerCP (clone SK7), anti-CD4-APC (clone SK3), anti-CD4-PE-Cy7 (clone SK3), anti- CD8-APC-H7 (clone SK1), anti-CD25-PE (clone M-A251), anti-CD28-APC (clone CD28.2), anti-CD38-APC (clone HIT2), anti-CD45RA-FITC (clone L48), anti-CD45RA-PE (clone HI100), anti-CD45RO-PE

(clone UCHL-1), anti-CD69-FITC (clone FN50), anti-CD57-FITC (clone NK-1), anti-CD195-PE-Cy7 (clone 2D7/CCR5), anti-CD197-PE-Cy7 (clone 3D12), anti-TCRa/b-FITC (clone T10B9.1A-31), anti-TCRg/d-PE (clone11F2), anti-CD14-APC-H7 (clone MΦP9), anti-CD16-PE (clone 3G8), anti-CD11b/Mac-1-Pacific Blue (clone ICRF44), anti-CD11c-APC (clone B-LY6), anti-human leukocyte antigen (HLA)-DR-PE-Cy7 (L243) (BD, Pharmingen, CA, USA). After staining, cells were washed twice with PBS containing 1% FBS. Cells were processed in the FACS Canto II or the FACSVerse flow cytometer (Becton Dickinson, CA, USA) and analyzed using FlowJo software (TreeStar, USA). Lymphocytes were gated according to forward scatter (FSC) and side scatter (SSC) signals from PBMC and T cells were gated according CD3 expression. Percentages of CD4, CD8, and Double Negative T cells (DN-T) were assessed. Each of the subpopulations was next analyzed for presence of naïve (CD45RA+CCR7+), central memory (CD45RA-CCR7+), effector memory (CD45RA-CCR7-) and CD45RA+ effector (CD45RA+CCR7-) cells. In T cells and their subsets the expressions of surface activation markers were then assessed. A panel of circulating blood cell features was performed using FACSVerse flow cytometer (Becton Dickinson, CA, USA) and FlowJo software (TreeStar, USA) as previously described [12, 14-16]. In brief, lymphocytes were gated according to forward scatter (FSC) and side scatter (SSC) signals from PBMC and T cells were gated according CD3 expression. Percentages of CD4, CD8, and Double Negative T cells (DN-T) were assessed. Next, each of the subpopulations was analysed for presence of naïve (CD45RA+CCR7+), central memory (CD45RA-CCR7+), effector memory (CD45RA-CCR7-) and CD45RA+ effector (CD45RA+CCR7-) cells. In T cells and their subsets, the expressions of surface activation markers were then assessed. Monocytes were gated according to forward scatter (FSC) and side scatter (SSC) signals as described previously [12, 14, 15]. Subsequently, cells were gated in an HLA-DR/CD14 plot to exclude HLA-DR-negative Natural Killer cells. Finally, monocyte subsets were defined according to the CD14 and CD16 expression, which allowed for discrimination of major monocyte subpopulations: CD14++CD16−, CD14++CD16+ and CD14+CD16++. Fluorescence Minus One (FMO) controls were used to determine the positivity of evaluated antigens. Data were visualized using FlowJo and heat map plots generated by ClustVis software [17].

Antibody conjugated with fluorochrome	Clone	Company
CD3-PerCP	SK7	BD Pharmingen
CD4-APC	SK3	BD Pharmingen
CD4-PE-Cy7	SK3	BD Pharmingen
CD8-APC-H7	SK1	BD Pharmingen
CD25-PE	M-A251	BD Pharmingen
CD28-APC	CD28.2	BD Pharmingen
CD38-APC	HIT2	BD Pharmingen
CD45RA-FITC	L48	BD Pharmingen
CD45RA-PE	HI100	BD Pharmingen
CD45RO-PE	UCHL-1	BD Pharmingen
CD69-FITC	FN50	BD Pharmingen
CD57-FITC	NK-1	BD Pharmingen
CD195-PE-Cy7	2D7/CCR5	BD Pharmingen
CD197-PE-Cy7	3D12	BD Pharmingen
TCR a/b-FITC	T10B9.1A-31	BD Pharmingen
TCR g/d-PE	11F2	BD Pharmingen
CD14-APC-H7	MΦP9	BD Pharmingen
CD16-PE	3G8	BD Pharmingen
CD11B/Mac-1-Pacific Blue	ICRF44	BD Pharmingen
CD11c-APC	B-LY6	BD Pharmingen
HLA-DR- PE-Cy7	L243	BD Pharmingen

Plasma cytokine measurements

Blood samples were centrifuged at 400 x*g* for 10 min. Then, platelet-rich plasma was collected and centrifuged at 1000 xg for 15 min at 4°C. Next, plasma sample without any pelleted cells were collected and stored at -80°C until analysis. Samples were analyzed for IFN-γ, IL-1β, IL-6, IL-10, IL-17A, IL-17E, IL-23, IL-33, MIP-3α/CCL20 and TNF-α with Luminex technology using standard kits with magnetic beads (MILLIPLEX MAP Human TH17 Panel - Immunology Multiplex Assay HTH17MAG-14K, Millipore, Merck) and were read on a Luminex 200 machine (Biorad) in accordance with the manufacturer's instructions.

Statistical analysis

Analyses were performed with SPSS (ver. 25.0) statistical package, unless otherwise stated. MR analysis was performed using MR-PRESSO (Mendelian Randomization Pleiotropy RESidual Sum and Outlier) [2]. Additional causal estimation analyses were performed using inverse variance-weighted (IVW), and simple median-based methods using MendelianRandomization package in R (ver. 3.5.1). [18]

Based on previous evidence, the clinical study was powered (80%) with a sample of 50 participants per group to detect a 7 mmHg difference in blood pressure between study groups and a 13 mmHg standard deviation of the change in systolic blood pressure [19-22]. All the analyses were performed on the basis of the intention-to-treat principle, per protocol analyses for the outcomes reported are included. Means and 95% confidence intervals (95% C.I.)of continuous variables are presented according to treatment groups. Categorical variables counts and percentages are presented according to the treatment group and values were tested using chi-square test. In the discovery analyses cytokines and cell types were compared using paired t test for each of the treatment groups separately. Between group differences and differences from baseline to two-months follow up were tested with the use of repeated measures ANOVA with interaction term between group and time defined by 2 visits. We used one sided tests for analysis of cytokines and cell types based on hypothesis from previous studies [19]. Correlation analyses between the blood pressure outcomes and changes in dental parameters were performed using Spearman Rank tests. Mediation analysis was performed using mediation package in R [23, 24]and tested average causal mediation effect of treatment group on change in SBP that is due to the change in PPD. P values < 0.05 were considered significant.

Suppl. Table 1: Characteristics of SNPs selected as instrumental variables for Mendelian Randomization analysis

SNP	Locus	EA	NEA	EAF ICBP+ UKB	SBP			DBP			PP			Periodontitis				Ref
					Beta	SE	p	Beta	SE	p	Beta	SE	p	OR	95% C.I.	Nca/ Ncon	EAF	
rs729876	*LOC 107984137*	T	C	0.80	0.029	0.038	4.49E-01	0.005	0.022	8.06E-01	0.031	0.026	2.2E-01	1.24	1.15 1.34	5095/ 9908	0.79-0.80*	3
rs4284742	*SIGLEC5*	A	G	0.24	-0.059	0.037	1.09E-01	-0.022	0.021	2.94E-01	-0.032	0.025	2.1E-01	0.75	0.68 0.83	2027/ 8330	0.24	25
rs16870060	*MTND1P5*	T	G	0.09	-0.054	0.053	3.02E-01	-0.027	0.030	3.63E-01	-0.018	0.036	6.1E-0.1	0.74	0.66 0.81	4924/ 7301	0.09-0.11*	3
rs2738058	*DEFA1A3*	T	C	0.44	0.044	0.031	1.53E-01	0.018	0.018	2.94E-01	0.030	0.021	1.5E-0.1	1.28	1.18 1.38	2067/ 8533	0.43	25

EA=effect allele; NEA=non-effect allele, EAF=effect allele frequency; Nca=number of cases; Ncon=number of control subjects;
*range of EAF in control subpopulations

Supplementary Table 2. Activity (daytime) and Night average blood pressure phenotypes at baseline and 2 months after treatment. * p<0.05 vs Baseline

		CPT		IPT	
		**Baseline**	**2-months**	**Baseline**	**2-months**
**Activity**	**SBP**	**135.5±15.8**	**139.2±19.4**	**138.66±13.63**	**132.80±10.33***
	**DBP**	**82.8±9.7**	**84.8±10.8**	**87.54±8.76**	**84.20±8.80***
	**PP**	**51.7±10.8**	**54.5±13.2**	**50.02±8.69**	**47.75±6.19***
**Night**	**SBP**	**119.2±17.6**	**123.7±20.3**	**122.76±15.98**	**115.52±11.15***
	**DBP**	**70.2±11.1**	**72.2±11.9**	**73.42±10.03**	**69.83±7.95***
	**PP**	**48.9±9.8**	**51.6±12.4**	**47.78±8.75**	**45.23±7.61**

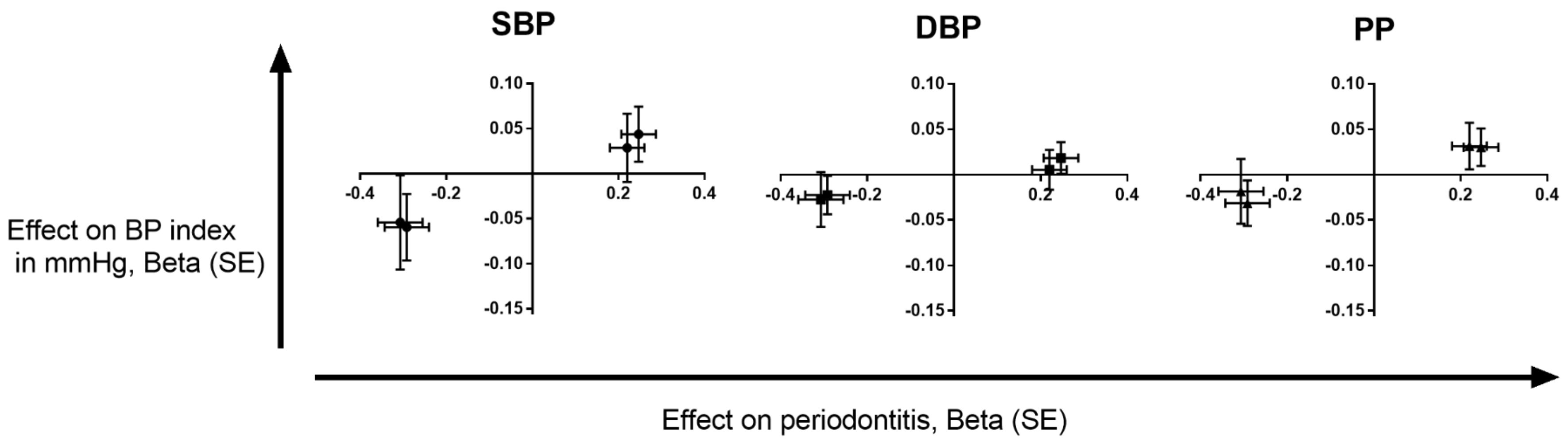

Suppl Figure S1. Effect sizes for each of the SNP genetic associations studied using Mendelian Randomization. Depicted points correspond to 4 SNPs used in MR analysis, i.e. from the most negative periodontitis Beta estimate (left to right): rs16870060 (effect allele=T), rs4284742 (A), rs729876 (T), rs2738058 (T) respectively.

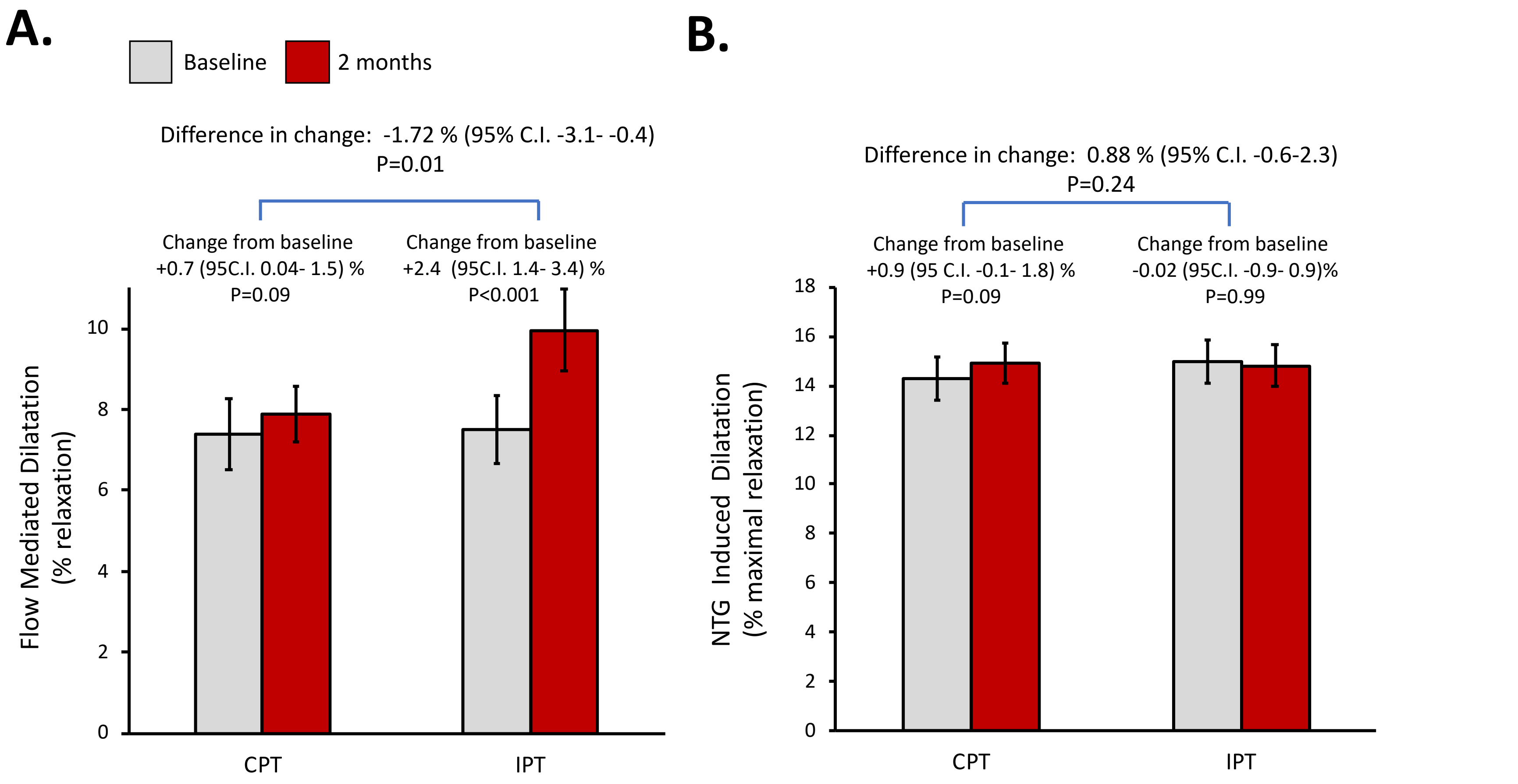

Suppl Figure S2. Effects of conventional (CPT) and intensive periodontal therapy (IPT) on vascular function. Changes of endothelium dependent (***Panel A***) and endothelium independent (***Panel B***) vasorelaxations between baseline and 2 months following CPT or IPT are reported as mean +/- 95% CI along with difference in change between randomization groups (with 95% CI).

Baseline characteristics	CPT n	IPT n	P value for interaction
Age ≥ 58 yrs	28	20	0.03
Age < 58 yrs	18	25	
Males	27	25	0.20
Females	19	20	
BMI > 28.3	24	22	0.09
BMI < 28.3	22	23	
PPD ≥ 3.02 mm	23	23	0.87
PPD < 3.02 mm	23	22	
CAL > 3.6 mm	24	21	0.60
CAL ≤ 3.6 mm	22	24	
CPITN > 3.3	25	19	0.67
CPITN ≤ 3.3	21	26	
a/h medications used > 2	18	17	0.19
a/h medications used ≤ 2	28	28	

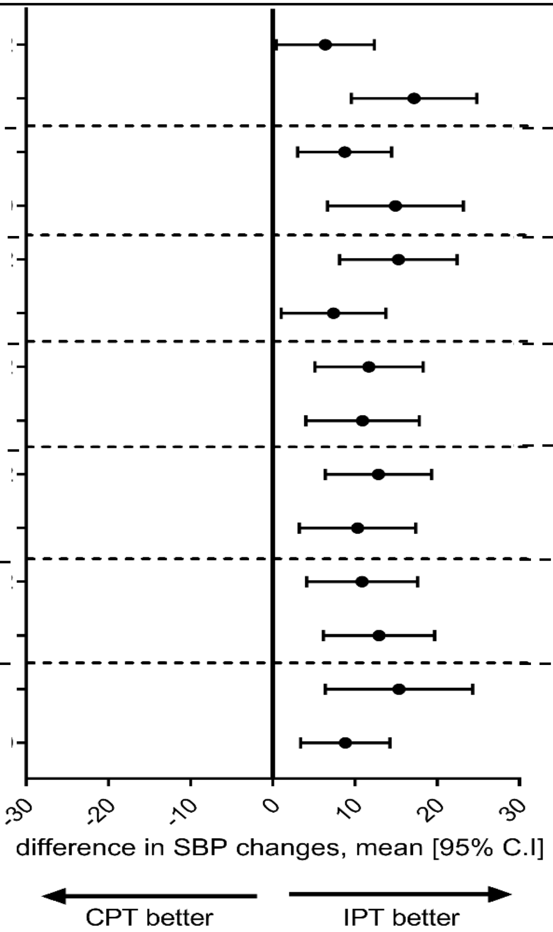

Suppl Figure S4. Subgroup analysis of the effects of CPT and IPT on systolic blood pressure in relation to baseline characteristics. Baseline characteristics included age, sex, BMI as well as periodontal status (PPD, CAL and CIPTN) and number of anti-hypertensive medications used. Patients were divided into subgroups according to median values.

References

1. Davies NM, Holmes MV, Davey Smith G. Reading Mendelian randomisation studies: a guide, glossary, and checklist for clinicians. BMJ 2018;**362**:k601.
2. Verbanck M, Chen CY, Neale B, Do R. Detection of widespread horizontal pleiotropy in causal relationships inferred from Mendelian randomization between complex traits and diseases. Nat Genet 2018;**50**(5):693-698.
3. Munz M, Richter GM, Loos BG, Jepsen S, Divaris K, Offenbacher S, Teumer A, Holtfreter B, Kocher T, Bruckmann C, Jockel-Schneider Y, Graetz C, Ahmad I, Staufenbiel I, van der Velde N, Uitterlinden AG, de Groot L, Wellmann J, Berger K, Krone B, Hoffmann P, Laudes M, Lieb W, Franke A, Erdmann J, Dommisch H, Schaefer AS. Meta-analysis of genome-wide association studies of aggressive and chronic periodontitis identifies two novel risk loci. Eur J Hum Genet 2019;**27**(1):102-113.
4. Munz M, Willenborg C, Richter GM, Jockel-Schneider Y, Graetz C, Staufenbiel I, Wellmann J, Berger K, Krone B, Hoffmann P, van der Velde N, Uitterlinden AG, de Groot L, Sawalha AH, Direskeneli H, Saruhan-Direskeneli G, Guzeldemir-Akcakanat E, Keceli HG, Laudes M, Noack B, Teumer A, Holtfreter B, Kocher T, Eickholz P, Meyle J, Doerfer C, Bruckmann C, Lieb W, Franke A, Schreiber S, Nohutcu RM, Erdmann J, Loos BG, Jepsen S, Dommisch H, Schaefer AS. A genome-wide association study identifies nucleotide variants at SIGLEC5 and DEFA1A3 as risk loci for periodontitis. Hum Mol Genet 2018;**27**(5):941-942.
5. Schaefer AS, Richter GM, Nothnagel M, Manke T, Dommisch H, Jacobs G, Arlt A, Rosenstiel P, Noack B, Groessner-Schreiber B, Jepsen S, Loos BG, Schreiber S. A genome-wide association study identifies GLT6D1 as a susceptibility locus for periodontitis. Hum Mol Genet 2010;**19**(3):553-62.
6. Evangelou E, Warren HR, Mosen-Ansorena D, Mifsud B, Pazoki R, Gao H, Ntritsos G, Dimou N, Cabrera CP, Karaman I, Ng FL, Evangelou M, Witkowska K, Tzanis E, Hellwege JN, Giri A, Velez Edwards DR, Sun YV, Cho K, Gaziano JM, Wilson PWF, Tsao PS, Kovesdy CP, Esko T, Magi R, Milani L, Almgren P, Boutin T, Debette S, Ding J, Giulianini F, Holliday EG, Jackson AU, Li-Gao R, Lin WY, Luan J, Mangino M, Oldmeadow C, Prins BP, Qian Y, Sargurupremraj M, Shah N, Surendran P, Theriault S, Verweij N, Willems SM, Zhao JH, Amouyel P, Connell J, de Mutsert R, Doney ASF, Farrall M, Menni C, Morris AD, Noordam R, Pare G, Poulter NR, Shields DC, Stanton A, Thom S, Abecasis G, Amin N, Arking DE, Ayers KL, Barbieri CM, Batini C, Bis JC, Blake T, Bochud M, Boehnke M, Boerwinkle E, Boomsma DI, Bottinger EP, Braund PS, Brumat M, Campbell A, Campbell H, Chakravarti A, Chambers JC, Chauhan G, Ciullo M, Cocca M, Collins F, Cordell HJ, Davies G, de Borst MH, de Geus EJ, Deary IJ, Deelen J, Del Greco MF, Demirkale CY, Dorr M, Ehret GB, Elosua R, Enroth S, Erzurumluoglu AM, Ferreira T, Franberg M, Franco OH, Gandin I, Gasparini P, Giedraitis V, Gieger C, Girotto G, Goel A, Gow AJ, Gudnason V, Guo X, Gyllensten U, Hamsten A, Harris TB, Harris SE, Hartman CA, Havulinna AS, Hicks AA, Hofer E, Hofman A, Hottenga JJ, Huffman JE, Hwang SJ, Ingelsson E, James A, Jansen R, Jarvelin MR, Joehanes R, Johansson A, Johnson AD, Joshi PK, Jousilahti P, Jukema JW, Jula A, Kahonen M, Kathiresan S, Keavney BD, Khaw KT, Knekt P, Knight J, Kolcic I, Kooner JS, Koskinen S, Kristiansson K, Kutalik Z, Laan M, Larson M, Launer LJ, Lehne B, Lehtimaki T, Liewald DCM, Lin L, Lind L, Lindgren CM, Liu Y, Loos RJF, Lopez LM, Lu Y, Lyytikainen LP, Mahajan A, Mamasoula C, Marrugat J,

Marten J, Milaneschi Y, Morgan A, Morris AP, Morrison AC, Munson PJ, Nalls MA, Nandakumar P, Nelson CP, Niiranen T, Nolte IM, Nutile T, Oldehinkel AJ, Oostra BA, O'Reilly PF, Org E, Padmanabhan S, Palmas W, Palotie A, Pattie A, Penninx B, Perola M, Peters A, Polasek O, Pramstaller PP, Nguyen QT, Raitakari OT, Ren M, Rettig R, Rice K, Ridker PM, Ried JS, Riese H, Ripatti S, Robino A, Rose LM, Rotter JI, Rudan I, Ruggiero D, Saba Y, Sala CF, Salomaa V, Samani NJ, Sarin AP, Schmidt R, Schmidt H, Shrine N, Siscovick D, Smith AV, Snieder H, Sober S, Sorice R, Starr JM, Stott DJ, Strachan DP, Strawbridge RJ, Sundstrom J, Swertz MA, Taylor KD, Teumer A, Tobin MD, Tomaszewski M, Toniolo D, Traglia M, Trompet S, Tuomilehto J, Tzourio C, Uitterlinden AG, Vaez A, van der Most PJ, van Duijn CM, Vergnaud AC, Verwoert GC, Vitart V, Volker U, Vollenweider P, Vuckovic D, Watkins H, Wild SH, Willemsen G, Wilson JF, Wright AF, Yao J, Zemunik T, Zhang W, Attia JR, Butterworth AS, Chasman DI, Conen D, Cucca F, Danesh J, Hayward C, Howson JMM, Laakso M, Lakatta EG, Langenberg C, Melander O, Mook-Kanamori DO, Palmer CNA, Risch L, Scott RA, Scott RJ, Sever P, Spector TD, van der Harst P, Wareham NJ, Zeggini E, Levy D, Munroe PB, Newton-Cheh C, Brown MJ, Metspalu A, Hung AM, O'Donnell CJ, Edwards TL, Psaty BM, Tzoulaki I, Barnes MR, Wain LV, Elliott P, Caulfield MJ, Million Veteran P. Genetic analysis of over 1 million people identifies 535 new loci associated with blood pressure traits. Nat Genet 2018;**50**(10):1412-1425.

7. International Consortium for Blood Pressure Genome-Wide Association S, Ehret GB, Munroe PB, Rice KM, Bochud M, Johnson AD, Chasman DI, Smith AV, Tobin MD, Verwoert GC, Hwang SJ, Pihur V, Vollenweider P, O'Reilly PF, Amin N, Bragg-Gresham JL, Teumer A, Glazer NL, Launer L, Zhao JH, Aulchenko Y, Heath S, Sober S, Parsa A, Luan J, Arora P, Dehghan A, Zhang F, Lucas G, Hicks AA, Jackson AU, Peden JF, Tanaka T, Wild SH, Rudan I, Igl W, Milaneschi Y, Parker AN, Fava C, Chambers JC, Fox ER, Kumari M, Go MJ, van der Harst P, Kao WH, Sjogren M, Vinay DG, Alexander M, Tabara Y, Shaw-Hawkins S, Whincup PH, Liu Y, Shi G, Kuusisto J, Tayo B, Seielstad M, Sim X, Nguyen KD, Lehtimaki T, Matullo G, Wu Y, Gaunt TR, Onland-Moret NC, Cooper MN, Platou CG, Org E, Hardy R, Dahgam S, Palmen J, Vitart V, Braund PS, Kuznetsova T, Uiterwaal CS, Adeyemo A, Palmas W, Campbell H, Ludwig B, Tomaszewski M, Tzoulaki I, Palmer ND, consortium CA, Consortium CK, KidneyGen C, EchoGen c, consortium C-H, Aspelund T, Garcia M, Chang YP, O'Connell JR, Steinle NI, Grobbee DE, Arking DE, Kardia SL, Morrison AC, Hernandez D, Najjar S, McArdle WL, Hadley D, Brown MJ, Connell JM, Hingorani AD, Day IN, Lawlor DA, Beilby JP, Lawrence RW, Clarke R, Hopewell JC, Ongen H, Dreisbach AW, Li Y, Young JH, Bis JC, Kahonen M, Viikari J, Adair LS, Lee NR, Chen MH, Olden M, Pattaro C, Bolton JA, Kottgen A, Bergmann S, Mooser V, Chaturvedi N, Frayling TM, Islam M, Jafar TH, Erdmann J, Kulkarni SR, Bornstein SR, Grassler J, Groop L, Voight BF, Kettunen J, Howard P, Taylor A, Guarrera S, Ricceri F, Emilsson V, Plump A, Barroso I, Khaw KT, Weder AB, Hunt SC, Sun YV, Bergman RN, Collins FS, Bonnycastle LL, Scott LJ, Stringham HM, Peltonen L, Perola M, Vartiainen E, Brand SM, Staessen JA, Wang TJ, Burton PR, Soler Artigas M, Dong Y, Snieder H, Wang X, Zhu H, Lohman KK, Rudock ME, Heckbert SR, Smith NL, Wiggins KL, Doumatey A, Shriner D, Veldre G, Viigimaa M, Kinra S, Prabhakaran D, Tripathy V, Langefeld CD, Rosengren A, Thelle DS, Corsi AM, Singleton A, Forrester T, Hilton G, McKenzie CA, Salako T, Iwai N, Kita Y, Ogihara T, Ohkubo T, Okamura T, Ueshima H, Umemura S, Eyheramendy S, Meitinger T, Wichmann HE, Cho YS, Kim HL, Lee JY, Scott J, Sehmi JS, Zhang W, Hedblad B, Nilsson P, Smith GD, Wong A, Narisu N, Stancakova

A, Raffel LJ, Yao J, Kathiresan S, O'Donnell CJ, Schwartz SM, Ikram MA, Longstreth WT, Jr., Mosley TH, Seshadri S, Shrine NR, Wain LV, Morken MA, Swift AJ, Laitinen J, Prokopenko I, Zitting P, Cooper JA, Humphries SE, Danesh J, Rasheed A, Goel A, Hamsten A, Watkins H, Bakker SJ, van Gilst WH, Janipalli CS, Mani KR, Yajnik CS, Hofman A, Mattace-Raso FU, Oostra BA, Demirkan A, Isaacs A, Rivadeneira F, Lakatta EG, Orru M, Scuteri A, Ala-Korpela M, Kangas AJ, Lyytikainen LP, Soininen P, Tukiainen T, Wurtz P, Ong RT, Dorr M, Kroemer HK, Volker U, Volzke H, Galan P, Hercberg S, Lathrop M, Zelenika D, Deloukas P, Mangino M, Spector TD, Zhai G, Meschia JF, Nalls MA, Sharma P, Terzic J, Kumar MV, Denniff M, Zukowska-Szczechowska E, Wagenknecht LE, Fowkes FG, Charchar FJ, Schwarz PE, Hayward C, Guo X, Rotimi C, Bots ML, Brand E, Samani NJ, Polasek O, Talmud PJ, Nyberg F, Kuh D, Laan M, Hveem K, Palmer LJ, van der Schouw YT, Casas JP, Mohlke KL, Vineis P, Raitakari O, Ganesh SK, Wong TY, Tai ES, Cooper RS, Laakso M, Rao DC, Harris TB, Morris RW, Dominiczak AF, Kivimaki M, Marmot MG, Miki T, Saleheen D, Chandak GR, Coresh J, Navis G, Salomaa V, Han BG, Zhu X, Kooner JS, Melander O, Ridker PM, Bandinelli S, Gyllensten UB, Wright AF, Wilson JF, Ferrucci L, Farrall M, Tuomilehto J, Pramstaller PP, Elosua R, Soranzo N, Sijbrands EJ, Altshuler D, Loos RJ, Shuldiner AR, Gieger C, Meneton P, Uitterlinden AG, Wareham NJ, Gudnason V, Rotter JI, Rettig R, Uda M, Strachan DP, Witteman JC, Hartikainen AL, Beckmann JS, Boerwinkle E, Vasan RS, Boehnke M, Larson MG, Jarvelin MR, Psaty BM, Abecasis GR, Chakravarti A, Elliott P, van Duijn CM, Newton-Cheh C, Levy D, Caulfield MJ, Johnson T. Genetic variants in novel pathways influence blood pressure and cardiovascular disease risk. Nature 2011;**478**(7367):103-9.

8. Mancia G, De Backer G, Dominiczak A, Cifkova R, Fagard R, Germano G, Grassi G, Heagerty AM, Kjeldsen SE, Laurent S, Narkiewicz K, Ruilope L, Rynkiewicz A, Schmieder RE, Struijker Boudier HA, Zanchetti A, Vahanian A, Camm J, De Caterina R, Dean V, Dickstein K, Filippatos G, Funck-Brentano C, Hellemans I, Kristensen SD, McGregor K, Sechtem U, Silber S, Tendera M, Widimsky P, Zamorano JL, Kjeldsen SE, Erdine S, Narkiewicz K, Kiowski W, Agabiti-Rosei E, Ambrosioni E, Cifkova R, Dominiczak A, Fagard R, Heagerty AM, Laurent S, Lindholm LH, Mancia G, Manolis A, Nilsson PM, Redon J, Schmieder RE, Struijker-Boudier HA, Viigimaa M, Filippatos G, Adamopoulos S, Agabiti-Rosei E, Ambrosioni E, Bertomeu V, Clement D, Erdine S, Farsang C, Gaita D, Kiowski W, Lip G, Mallion JM, Manolis AJ, Nilsson PM, O'Brien E, Ponikowski P, Redon J, Ruschitzka F, Tamargo J, van Zwieten P, Viigimaa M, Waeber B, Williams B, Zamorano JL, The task force for the management of arterial hypertension of the European Society of H, The task force for the management of arterial hypertension of the European Society of C. 2007 Guidelines for the management of arterial hypertension: The Task Force for the Management of Arterial Hypertension of the European Society of Hypertension (ESH) and of the European Society of Cardiology (ESC). Eur Heart J 2007;**28**(12):1462-536.
9. Williams B, Mancia G, Spiering W, Agabiti Rosei E, Azizi M, Burnier M, Clement DL, Coca A, de Simone G, Dominiczak A, Kahan T, Mahfoud F, Redon J, Ruilope L, Zanchetti A, Kerins M, Kjeldsen SE, Kreutz R, Laurent S, Lip GYH, McManus R, Narkiewicz K, Ruschitzka F, Schmieder RE, Shlyakhto E, Tsioufis C, Aboyans V, Desormais I, Group ESCSD. 2018 ESC/ESH Guidelines for the management of arterial hypertension. Eur Heart J 2018;**39**(33):3021-3104.

10. Page RC, Eke PI. Case definitions for use in population-based surveillance of periodontitis. J Periodontol 2007;**78**(7 Suppl):1387-99.

11. Eke PI, Page RC, Wei L, Thornton-Evans G, Genco RJ. Update of the case definitions for population-based surveillance of periodontitis. J Periodontol 2012;**83**(12):1449-54.

12. Mikolajczyk TP, Osmenda G, Batko B, Wilk G, Krezelok M, Skiba D, Sliwa T, Pryjma JR, Guzik TJ. Heterogeneity of peripheral blood monocytes, endothelial dysfunction and subclinical atherosclerosis in patients with systemic lupus erythematosus. Lupus 2016;**25**(1):18-27.

13. Wilk G, Osmenda G, Matusik P, Nowakowski D, Jasiewicz-Honkisz B, Ignacak A, Czesnikiewicz-Guzik M, Guzik TJ. Endothelial function assessment in atherosclerosis: comparison of brachial artery flowmediated vasodilation and peripheral arterial tonometry. Pol Arch Med Wewn 2013;**123**(9):443-52.

14. Itani HA, McMaster WG, Jr., Saleh MA, Nazarewicz RR, Mikolajczyk TP, Kaszuba AM, Konior A, Prejbisz A, Januszewicz A, Norlander AE, Chen W, Bonami RH, Marshall AF, Poffenberger G, Weyand CM, Madhur MS, Moore DJ, Harrison DG, Guzik TJ. Activation of Human T Cells in Hypertension: Studies of Humanized Mice and Hypertensive Humans. Hypertension 2016;**68**(1):123-32.

15. Loperena R, Van Beusecum JP, Itani HA, Engel N, Laroumanie F, Xiao L, Elijovich F, Laffer CL, Gnecco JS, Noonan J, Maffia P, Jasiewicz-Honkisz B, Czesnikiewicz-Guzik M, Mikolajczyk T, Sliwa T, Dikalov S, Weyand CM, Guzik TJ, Harrison DG. Hypertension and increased endothelial mechanical stretch promote monocyte differentiation and activation: roles of STAT3, interleukin 6 and hydrogen peroxide. Cardiovasc Res 2018;**114**(11):1547-1563.

16. Maga P, Mikolajczyk TP, Partyka L, Siedlinski M, Maga M, Krzanowski M, Malinowski K, Luc K, Nizankowski R, Bhatt DL, Guzik TJ. Involvement of CD8+ T cell subsets in early response to vascular injury in patients with peripheral artery disease in vivo. Clin Immunol 2018;**194**:26-33.

17. Metsalu T, Vilo J. ClustVis: a web tool for visualizing clustering of multivariate data using Principal Component Analysis and heatmap. Nucleic Acids Res 2015;**43**(W1):W566-70.

18. Yavorska O, Staley J. MendelianRandomization: Mendelian Randomization Package. R package version 0.4.1. 2019.

19. D'Aiuto F, Gkranias N, Bhowruth D, Khan T, Orlandi M, Suvan J, Masi S, Tsakos G, Hurel S, Hingorani AD, Donos N, Deanfield JE, Group T. Systemic effects of periodontitis treatment in patients with type 2 diabetes: a 12 month, single-centre, investigator-masked, randomised trial. Lancet Diabetes Endocrinol 2018;**6**(12):954-965.

20. Mansoor GA. Ambulatory blood pressure monitoring in clinical trials in adults and children. Am J Hypertens 2002;**15**(2 Pt 2):38S-42S.

21. Osmenda G, Maciag J, Wilk G, Maciag A, Nowakowski D, Loster J, Dembowska E, Robertson D, Guzik T, Czesnikiewicz-Guzik M. Treatment of denture-related stomatitis improves endothelial function assessed by flow-mediated vascular dilation. Arch Med Sci 2017;**13**(1):66-74.

22. Symplicity HTNI, Esler MD, Krum H, Sobotka PA, Schlaich MP, Schmieder RE, Bohm M. Renal sympathetic denervation in patients with treatment-resistant hypertension (The Symplicity HTN-2 Trial): a randomised controlled trial. Lancet 2010;**376**(9756):1903-9.

23. Dustin Tingley D, Yamamoto T, Hirose K, Keele L, Imai K. mediation: R Package for Causal Mediation Analysis. . Journal of Statistical Software, 2014;**59(5), 1-38**:http://www.jstatsoft.org/v59/i05/.

24. Imai K, Keele L, Tingley D. A general approach to causal mediation analysis. Psychol Methods 2010;**15**(4):309-34.

25. Munz M, Willenborg C, Richter GM, Jockel-Schneider Y, Graetz C, Staufenbiel I, Wellmann J, Berger K, Krone B, Hoffmann P, van der Velde N, Uitterlinden AG, de Groot L, Sawalha AH, Direskeneli H, Saruhan-Direskeneli G, Guzeldemir-Akcakanat E, Keceli HG, Laudes M, Noack B, Teumer A, Holtfreter B, Kocher T, Eickholz P, Meyle J, Doerfer C, Bruckmann C, Lieb W, Franke A, Schreiber S, Nohutcu RM, Erdmann J, Loos BG, Jepsen S, Dommisch H, Schaefer AS. A genome-wide association study identifies nucleotide variants at SIGLEC5 and DEFA1A3 as risk loci for periodontitis. Hum Mol Genet 2017;**26**(13):2577-2588.
